# Resisting stigma: the role of online communities in young mothers’ successful breastfeeding

**DOI:** 10.1186/s13006-024-00626-z

**Published:** 2024-03-06

**Authors:** Christina Severinsen, Eva Neely, Rochelle Hutson

**Affiliations:** 1 School of Health Sciences, Massey University, Palmerston North, Aotearoa, New Zealand; 2 School of Health, Victoria University of Wellington, Wellington, Aotearoa New Zealand; 3 School of Health Sciences, Massey University, Palmerston North, Aotearoa, New Zealand

**Keywords:** Breastfeeding, Young mothers, Online, Social support, Stigma

## Abstract

**Background:**

Breastfeeding initiation and continuation rates are shaped by complex and interrelated determinants across individual, interpersonal, community, organisational, and policy spheres. Young mothers, however, face a double burden of stigma, being perceived as immature and incompetent in their mothering and breastfeeding abilities. In this study, we aimed to understand the experiences of young mothers who exclusively breastfed for six months and beyond and explore their experiences of stigma and active resistance through social media.

**Methods:**

In 2020, in-depth telephone interviews about breastfeeding experiences were conducted with 44 young mothers under age 25 in Aotearoa New Zealand who breastfed for six months or longer. Participants were recruited via social media. Interviews were audio-recorded, transcribed and analysed thematically.

**Results:**

Analysis yielded four themes on young mothers’ negotiation of breastfeeding and support. The first three themes revealed young mothers’ encounters with socio-cultural contexts. They faced negative judgments about maturity and competence, adverse guidance to supplement or cease breastfeeding, and an undermining of their breastfeeding efforts. The fourth theme showed how young mothers sought alternative support in online environments to avoid negative interactions. Online spaces provided anonymity, convenience, experiential knowledge and social connections with shared values. This facilitated identity strengthening, empowerment and stigma resistance.

**Conclusion:**

Our research highlights the importance of online communities as a tool for young mothers to navigate and resist the societal stigmas surrounding breastfeeding. Online spaces can provide a unique structure that can help counteract the adverse effects of social and historical determinants on breastfeeding rates by fostering a sense of inclusion and support. These findings have implications for the development of breastfeeding promotion strategies for young mothers and highlight the potential of peer support in counteracting the negative impacts of stigma. The research also sheds light on the experiences of young mothers within the health professional relationship and the effects of stigma and cultural health capital on their engagement and withdrawal from services. Further research should examine how sociocultural barriers to breastfeeding stigmatise and marginalise young mothers and continue to reflect on their socio-political and economic positioning and how it can exacerbate inequities.

## Background

Breastfeeding is complex and multi-faceted, influenced by individual, interpersonal, community, organisational, and policy environments [1–4]. Social disparities in breastfeeding duration have been identified, with certain groups, including young mothers, experiencing a higher risk of shorter breastfeeding duration [5–6]. These mothers may lack access to the necessary determinants of successful breastfeeding continuation [6–8]. To overcome these challenges, mothers need access to supportive social environments, education, services, and protective policies [3]. However, a lack of support, stigma, organisational practices, and national policies can undermine breastfeeding efforts [3, 6]. Social support, in particular, is a critical factor for breastfeeding intention, initiation, and continuation [9–13]. In Aotearoa New Zealand, despite a 96.8% rate of exclusive breastfeeding in the first month, this percentage sharply declines to 35.5% when infants reach six months of age [14]. Notably, maternal age is a significant contributing factor to these disparities. Young mothers aged under 25 years encounter significant challenges in adhering to the recommended practice of exclusive breastfeeding for up to six months. Only 19.3% of these babies are exclusively breastfed until they reach six months [14].

In recent years, many mothers have turned to online platforms for breastfeeding support, but there is little research on the role of social media in this area, especially for young mothers [12, 15–16]. Our research aims to address this gap by studying the experiences of young mothers under 25 who have successfully maintained breastfeeding over time. We also investigate the determinants of their success and the impact of healthcare on breastfeeding rates [17]. Previous breastfeeding research has primarily focused on majority groups and has not been conducted in Aotearoa New Zealand, or with young mothers [7, 18]. Additionally, young mothers are often portrayed negatively, and their breastfeeding capabilities are framed as ‘at risk’ and ‘vulnerable’ [19]. Our study aims to challenge these negative stereotypes by highlighting the successful breastfeeding journeys of these young mothers and the role of online social support.

### Online breastfeeding support

New mothers are increasingly using social media as their preferred method and first source of health advice and information for breastfeeding, particularly young mothers who are more digitally connected than their older peers [20–22]. This shift in information-seeking behaviour raises important questions about the role of traditional sources of breastfeeding support, such as health professionals, family, and books. While antenatal services can equip mothers with good breastfeeding knowledge, this awareness does not always translate into practice [18]. Formal breastfeeding information may be limited and contradictory, and mothers often express dissatisfaction with the practicality of information provided by health professionals [23 − 13]. Consequently, some mothers turn to social media and online fora due to perceived deficits in the breastfeeding support provided by health professionals, and other factors, such as accessibility and the sense of community, may also contribute to this trend [15, 18, 23].

Nevertheless, it is essential to acknowledge that online environments are not immune to misinformation. Promoting health literacy among young mothers is crucial to developing their knowledge and critical thinking skills, enabling them to assess the reliability of information and make informed decisions about their breastfeeding practice. While online platforms can offer valuable support and information, they cannot completely replace healthcare. Therefore, striking a balance between leveraging the benefits of online communities on social media platforms and ensuring that mothers receive evidence-based guidance and professional medical advice when needed is key. Failure to address this unmet need for practical breastfeeding guidance can lead to young mothers experiencing feelings of doubt, frustration, overwhelm, and isolation, ultimately undermining their confidence to succeed in breastfeeding [13, 15].

In online fora, such as private and public Facebook groups, mothers share their breastfeeding experiences with other breastfeeding mothers as peers, and the access to shared knowledge and authentic, real-world experiences is often trusted and highly valued [10, 24]. Online fora allow mothers to access valuable information to enhance their understanding throughout their breastfeeding journeys, including milk production, nutrition, and positioning [25]. These spaces allow for user-created content and reciprocal sharing and receiving of advice, and mothers use online groups to triangulate information from a range of informal and formal sources [23, 26]. Online sources of breastfeeding support are also experienced as convenient, fast, flexible, and responsive. Easy connection to one-off and ongoing support in a timely and accessible manner is a crucial motivator for accessing breastfeeding help online [23, 27–28]. The convenience of online information and guidance removes barriers such as logistics and physical access to services [28, 29]. There is evidence that accessibility is particularly important for young mothers and is an important means of communicating with their social networks [30].

Several researchers have found that online advice, reassurance, and learning are linked to continued breastfeeding. Engagement in online spaces during the transition to motherhood enhances confidence and provides emotional support, reassurance, and solidarity [23, 28, 31, 32]. Accessing online information and support contributes positively to breastfeeding rates by improving confidence in public breastfeeding, increasing intention and motivation to breastfeed longer, and normalising breastfeeding [23, 24, 28, 31]. These positive experiences are particularly significant for geographically isolated mothers and those experiencing breastfeeding challenges [15].

### Stigma and young mothers

Goffman [33] defined stigma as “blemishes of individual character” that discredit an individual in their social setting. Stigma is shaped by societal expectations and moral attitudes, with teenage pregnancy and parenting often stigmatised due to negative evaluations and assumptions of promiscuity, irresponsibility, and dependency [12, 34, 35]. However, such stereotypes and associations are overstated and fail to acknowledge the impact of societal inequalities and stigmatisation on the wellbeing of teenage mothers [34, 36].

A significant amount of research has documented the frequent experiences of stigma, judgment, and discrimination faced by young mothers [7, 12, 34, 36]. Young mothers are often stigmatised as “bad mothers” solely based on their age [34, 35]. They are judged for factors such as being undesirable, immature, unemployed, living in poverty, having low education levels, having behaviour problems, having poor parenting abilities, and having low career potential [34, 37]. The participants also experience stigma through verbal and non-verbal expressions of poor treatment, such as surveillance, dismissive, scorn, discrimination, and cold behaviour, often experienced in healthcare interactions [12, 35, 36]. Many young mothers also fear being stigmatised by others and revealing their status as young mothers [12].

The challenges faced by young mothers related to breastfeeding, such as latching, cluster feeding, and discomfort with breastfeeding in public, are not unique to them but instead are shared among all mothers [38]. All mothers may experience judgment and stigmatisation regarding their parenting and feeding practices. Ellis-Sloan notes that societal expectations of “good” motherhood place high demands on all women [34], and strong associations are made between “good mothering” and breastfeeding [39–40], while formula feeding carries a stigma and can lead to shame [38]. These societal expectations create a moral imperative for breastfeeding, leading to scrutiny and intervention of mothers and their decisions, bodies and experiences [38]. For young mothers, their age is often used as a means to discredit them as “good mothers” and “breastfeeding mothers”, and they may face additional pressure to positively portray themselves and their parenting practices in the face of societal stigma against teenage parenthood [34].

Research has shown that young mothers often face additional challenges regarding breastfeeding, such as limited access to support services, marginalisation, and societal judgments [7, 18]. These challenges are compounded by societal perceptions of young mothers as incompetent parents and the stigma associated with breastfeeding [12, 38]. Some researchers describe this double stigma, where young mothers are stigmatised based on their age and breastfeeding, as “deviant” behaviour [7, 12, 37]. In the research by Jamie et al., young mothers described how breastfeeding itself required justification as questionable behaviour due to societal and cultural norms [38]. They can face additional barriers, such as social and financial capital and education levels [41–43].

The normative expectations that young mothers will not breastfeed their babies are related to tension in relationships with health practitioners, resulting from mothers feeling undermined and ignored [37]. In their meta-analysis of breastfeeding perceptions and experiences, Schmied et al. found that young mothers felt they were not generally encouraged or expected to breastfeed by health professionals [44]. They experience professional breastfeeding support as lacking and brief [12], and healthcare professionals do not expect or encourage them to initiate breastfeeding [43]. It has been found that health professionals often promote formula feeding to young mothers as a more normal and easier method than working through problems [12]. These mothers perceive this lack of support as connected to their age and a lack of expectations by the health professional [12]. They felt less favourable treatment and stigmatised because of their age: “Uneasy relationships between adolescent mothers and healthcare practitioners were… impacted by discriminatory assumptions about young mothers, power imbalances and ineffective communication” [37].

Understanding the role of inequities and social status in clinical relationships through the concept of cultural health capital provides valuable insights into the challenges faced by young mothers [45]. Cultural health capital, as defined by Dubbin et al., refers to “a specialised collection of cultural skills, attitudes, behaviours, and interactional styles that are valued, leveraged, and exchanged by both patients and providers during clinical interactions” [46] (p. 113). The stigma faced can erode cultural health capital in healthcare settings and reduce their status and power due to their positioning as young mothers. This relationship imbalance can compromise their ability to leverage cultural capital in their interactions with health professionals and others in their families and communities. Consequently, these mothers may feel shut down in interactions with health professionals, unable to benefit their breastfeeding practice through accessing care and participating in decision-making [37, 46].

Overall, the stigma surrounding young breastfeeding mothers negatively impacts their ability to continue breastfeeding. This stigma, which arises from societal and cultural norms, discourages them from seeking help and support from healthcare professionals and support services [30, 7, 34–35]. Breastfeeding challenges faced by young mothers can be exacerbated by stigma and low cultural health capital. When societal perceptions position young mothers as irresponsible and incompetent, this can deny them status, value and power. Low cultural health capital can compromise their ability to participate in clinical interactions and decision-making. Such negative attitudes and comments erode their confidence in their role as active participants in their care and as assertive mothers [12, 37]. Phoebe explains that these negative attitudes and discouraging comments during challenging times can cause doubt [15]. Ultimately, this stigma can lead them to avoid further interaction and perpetuate their social exclusion, creating barriers to accessing services and contributing to social isolation [35–36]. Chopel et al. link the “teen mom” stigma to lower rates of continued breastfeeding, as many young mothers change their feeding behaviours in response to unsupportive environments [7]. On the other hand, positive emotional and social support enables them to continue breastfeeding; however, stigma and low levels of support create significant difficulties in initiating, continuing, and exclusively breastfeeding for young mothers [7, 12, 18].

Given the stigmas faced, we were interested in talking to young mothers about breastfeeding journeys that did not end prematurely but extended to and beyond six months. Our project broadly aimed to learn about their determinants of success. We aimed to explore young mothers’ experiences of support, including within health services and online spaces, and how these experiences shaped their breastfeeding journey. This article examines the unhelpful advice, stigma, silencing, and judgement they have faced within their immediate social contexts. We will then show how these negative experiences have led to mistrust and disengagement from health services. These experiences can impact their access to healthcare services. We explore how young mothers have turned to online spaces to overcome breastfeeding challenges and receive support. We will examine the nature of online support and relationships and how they function as virtual communities, allowing them to challenge and resist negative stereotypes and discrimination. Through engaging in online communities, we argue that young breastfeeding mothers can effectively manage and resist stigma and discrimination.

## Method

We employed a qualitative, thematic analysis research method to investigate the experiences of young mothers who breastfed exclusively for six months or longer. The study focused on Aotearoa New Zealand mothers aged under 25 years of age who had breastfed their babies for at least six months. To recruit participants, we shared social media posts in seven active local Facebook parenting groups for mothers living in Aotearoa New Zealand. A total of 44 young mothers contacted the researchers to express interest. On average, these mothers were 21 years old (with a range of 18–24 years) when they had their first child and were 24 years old at the time of the interview. They had an average of 1.6 children each (with a range of 1–6 children). All 44 mothers agreed to participate in in-depth, semi-structured telephone interviews (average duration 44 min). Interviews were carried out by CS and EN. The research was explained to participants via an information sheet, and they provided written consent. During the interviews, participants were asked to reflect on various aspects of their breastfeeding journey, including their intentions, initiation, support, social and environmental influences, and the challenges and changes they experienced over time. The interviews were audio-recorded and transcribed for analysis.

We used thematic analysis to analyse the data and explore the meanings and representations of the determinants of successful breastfeeding [47–48]. This involved a reflective and thoughtful engagement with the collected data and identifying, analysing, and interpreting patterns of meaning. Thematic analysis was conducted recursively and flexibly, starting with a more inductive and explorative approach in the early stages and then moving to a more deductive approach in later stages, with a deeper engagement with relevant literature.

## Results

### Negative judgements as young breastfeeding mothers

In the course of discussing their breastfeeding journeys, all of the mothers described facing negative judgements related to their age and status as young mothers. While no direct questions were asked regarding stigma, their narratives emphasised frequent judgements and assumptions made about their competency, maturity and adequacy in the parenting role. Negative assumptions and stigma most often emerged when discussing interactions with health services, attempts to access support, and reactions from family members regarding their feeding decisions. They spoke of significant stigma relating to their identity as young mothers, characterised as disapproval, self-control failures and character blemishes.

I can remember being 21 and very visibly pregnant and getting that side eye from the older ladies. And that’s really not a great place to be in. (P31, 21 years).

Even with the lactation consultants… even they would judge you. There was a, ‘What right do you have to have a baby?’ ‘What must you have done to put yourself in this situation?’… My mum was telling me about these young mum homes that were 40, 50 years ago, and it was almost the same kind of stigma that would follow you… They keep saying, ‘A mistake child,’ and I’ve never viewed him as a mistake. That really upsets me; people saying that… some of those comments were completely unnecessary. I don’t think they would ever have said to me had I been in my early 30s like I was supposed to be, which was the socially acceptable thing.” (P32, 24 years).

Participants explained the scrutiny and opinions from others as challenging.

I definitely think when you’re younger, you’re more worried that you’re going to do something wrong. You’re used to other people being in authority, other people making the decisions. I think you’re also trying to prove to everyone that you can do it. So yeah, it takes a lot to either stand up to people if you disagree, or even to just… to not take what they’re saying as exactly right… and do what you want to do. (P35, 18 years).

The scrutiny and undermined agency participants described reflected a societal denial of the status and decision-making power afforded to older mothers.

Well, you might have the mothering instincts, but I found that people don’t necessarily believe that you do or think that you’re not old enough to know… You’re not trusted to buy alcohol, but you’ve got a baby, and… there’s a question of, ‘Oh, you’re not old enough to really make these decisions. (P30, 23 years).

The participants related the scrutiny to their undermined status as young mothers and difficulties in maintaining their agency in mothering. The perceptions and judgements of others were misaligned with their needs and self-identity. These judgements were described as unfair and unfounded. Participants’ narratives highlighted a refusal to accept conjectures about their ability to mother. The participants explained how judgements about their age and status as young mothers denied them the respect, legitimacy, and influence needed to assert themselves and their breastfeeding goals. Participants perceived these judgements as unfairly denying them the credibility and social value to claim authority regarding their own mothering abilities.

Massively isolating, ‘cos the parenting groups that you’d try to go to… you’d be judged. Everyone would think you’d slept around, and that’s how you ended up in the situation you’re in… Everyone was bottle feeding in the area we were in, just the demographic it was. So you were judged for not [formula feeding]. Yeah, everywhere you went, there was sort of that stigma of being a teenage parent; it seemed to paint you with a brush that was incredibly hard to break. People wouldn’t give you a chance, mainly because they’d automatically have these preconceived assumptions about you. (P32, 24 years).

The mothers also linked the stigma of young motherhood to ideas of the double or intersecting stigma of breastfeeding as a young mother:

There was a whole lot of judgement. Just the pure fact that I was a young mum, regardless of how I was feeding, we were judged for that, and then, yeah, add the breastfeeding on top of it, and I do think, attitudes. (P32, 24 years).

The stigma related to breastfeeding added to judgements on their ability. The participants reflected on narratives about young mothers ‘not being breastfeeders’ alongside normative social expectations to formula feed, particularly from health professionals.

[During] the visits to that nurse, I felt that was when I was being the most judged. My parenting, my age, everything about me and my baby were being judged. So I didn’t actually go for very long. I also remember having pressure to supplement, to start on formula really early, which I didn’t do… I didn’t want to do that. There was judgements about my capability… she didn’t feel I was old enough to be capable. I guess she had decided that I wasn’t going to [breastfeed]. (P22, 22 years).

The recurrence of feeling undermined by poor advice indicated an imbalance of power and status in relationships with health professionals. The participants’ accounts point to compromised cultural health capital in their interactions. The discrediting of their motivations to continue breastfeeding denied young mothers legitimacy and expertise over appropriate care. Their narratives show that young mothers experience stigma from the public and health professionals [34, 36].

### Adverse guidance and undermining of establishment and continuation of breastfeeding

Participants spoke of the widespread promotion of formula feeding right from birth. Being advised to supplement or cease breastfeeding was commonplace across the data, against the mother’s desires. Family members and health professionals often encouraged formula feeding in response to their perceptions of the demands on the mother and the baby’s sleep.

Family members said when it was quite difficult, they’re like, ‘Oh, you know, you should just give up and put her on the bottle,’ but I just felt strongly that I didn’t want to do that. (P26, 20 years).

However, for our participants, this guidance did not correspond with their breastfeeding goals and circumstances, potentially destabilising breastfeeding. For example, mothers spoke of frequent recommendations to supplement with formula despite knowing that introducing formula decreases supply. Such interventions by health professionals were not helpful to them. The dismissal and undermining of breastfeeding goals reflected participants’ low standing and eroded claims to capital and respect in clinical encounters. Unhelpful advice and the questioning of motivations to breastfeed stripped young mothers of legitimacy and power to seek appropriate care.

It’s interesting. Maternal Mental Health said, ‘Why don’t you express or put them on formula so [father] can do the night shift or take some sleeping pills so you can sleep?’ And I was like, ‘That’s not going to help. It would make two parents tired, and it’s not just the milk coming from my breast, it’s the comfort which is massive. (P34, 23 years).

They’ll just go, ‘Oh, just give him a little bit of formula.’ And then formula takes away from the mum making milk. So then, eventually, the formula ends up building up, and the milk goes down, and before you know it, the baby’s on formula whether the mother wanted to or not. When it comes down to it, they’ll do some sabotaging; they just won’t help as much as they should in terms of referring onwards [to lactation consultant]. (P33, 23 years).

The participants spoke of resisting formula as they were content with breastfeeding and often would have preferred to shift the focus of help towards other demands such as help with other children, meals and washing. There was often a sense that others felt that interjecting oneself into decisions around infant feeding was a helpful contribution, whilst the mothers felt such commentary undermined them. The mothers linked the promotion of formula feeding to prejudicial assumptions rooted in their denied status and value as young parents in the healthcare context.

I had people saying, ‘Oh, you need to give them some formula now.’ ‘Oh, people could help you if you gave them formula.’ But I would just say, ‘No, they’re not having formula. If people want to help, they can help in other ways, you know, like they can play with my [baby], or they can peg the washing, or they can change the baby’s bum, you know, give them a cuddle, literally do everything else but the feeding.’ And I thought, at the end of the day, people that are suggesting that I give them a formula bottle so other people can help, they’re not going to be the ones coming around at midnight to give them a formula bottle. It would still be me doing it, so it was pointless, you know? Like everyone suggests that, but it’s like, ‘Mate. You’re going to come around at midnight? Naha. (P28, 23 years).

The mothers spoke of advice received recurringly from family and health professionals as unhelpful and incorrect. Information was described as conflicting, inconsistent, and misaligned from their understanding. The mothers’ narratives highlighted their view of ongoing prejudice and ignorance about breastfeeding, particularly among health professionals. Breastfeeding support from health professionals was described as outdated and irrelevant.

I did see a Plunket^1^*nurse initially. She gave me some very bad advice around breastfeeding, which is why I stopped seeing her. I think she was quite old school. Her advice was, ‘Scrub your nipples with toothbrushes if you want to harden them up,’ and ‘Your baby will be overeating, and that’s why he’s spilling.’ All my children have been quite spilly. [Baby] was a particularly good gainer. He was one of those very chubby babies. She’d say that I was overfeeding him, or my milk was too calorie-dense, and I was overfeeding him, and I should restrict his feeds or consider supplementing him with something to prevent that. And then, because he was so big, she continued to suggest I should be giving him really runny rice stuff, Farex, from about three months because he was so big that my milk couldn’t possibly continue to nourish him. It’s that real old-school advice; I think my mum was probably given that kind of advice. I think the final straw came when he got teeth quite early too. She said that I had to make sure I was brushing his teeth after every breastfeed in the night because of the decay, and I was like, ‘Hang on, I’ve just learned about this at the peer counselling thing’, and I was like, ‘Oh no, hang on.‘”* (P30, 23 years)

There’s still lots of like doctors and others saying there’s no nutritional value in your milk, you know, ‘There’s no point in breastfeeding, just put them on cow’s milk,’ you still hear that kind of thing. (P22, 22 years).

The participants perceived others’ advice as unreliable, questioning the evidence base of health professional guidance.

I have had hyperemesis with some of them, and I went to the GP asking for medication, and she said that the medication was not compatible with breastfeeding. I said, ‘Ok, that’s fine, don’t give it to me if it’s not compatible with breastfeeding.’ And she then proceeded to tell me that there was no benefit to breastfeeding after six months, and it was all for the mother’s sake. And I walked out in tears… that was not obviously a medical opinion but a personal opinion. (P35, 18 years).

I didn’t like my Plunket Nurse. She was forever telling me my breastmilk wasn’t nutritious enough for my daughter. She was like her dad, tall and skinny, so she was really long, in the high percentile for her height. But then she was classified as ‘underweight’. My midwife would always say, ‘You should introduce her to this and that.’ She was not underweight, she’s nourished, and all that. (P16, 20 years).

Similarly, urging mothers to formula feed overnight was refuted by many participants.

I’ve heard, you know, people, ‘Aww, perhaps if you just gave them a bottle, then they’d sleep through the night’… Actually, they’re not really supposed to sleep through before two if you look at the research… But I did get a lot of pressure that perhaps they’d sleep through the night. (P15, 18 years).

Together, the stigma and unhelpful advice from people around them and how it could work to undermine their confidence had a clear impact on breastfeeding for themselves and other young mothers. Speaking in the third person, one mother explained her difficulties persisting with breastfeeding in the face of criticism.

I mean, if you had someone saying negative things about feeding all the time, it would be very hard to continue. Because it’s hard enough with a new baby without someone putting pressure on you in another way. (P13, 23 years).

### Employing strategies to manage and reduce stigma

Many young mothers in our study actively resisted stigma related to their breastfeeding practice It was evident that the confidence to speak up and ignore unhelpful and misleading advice was stronger with subsequent babies.

With [first baby], I listened to a lot of that advice. I actually night-weaned him at six months because somebody told me that I should. ‘At six months, he should be sleeping through the night,’ which is a load of rubbish anyway. I didn’t know any better. And the older generation giving helpful, ‘Aww, is he sleeping through yet?’ and that sort of thing, and, ‘Aww, he doesn’t need to feed again.’ You know, just little things that when you’re younger, it’s just little snide comments like, ‘Oh, he’s feeding again.’ And you’re like, ‘Yeah, I was ok with that,’ and then you go, ‘Ohh, well, maybe he shouldn’t be feeding again,’ sort of thing. It sort of just gets in your head a little bit. I think it takes a lot of work to fight that sort of stuff. It’s that trust. Once you’ve had a couple, you sort of start to get more like, ‘Ah, let’s just do it our way.’ But yeah, it is an impact with being young too. You’re wanting to do it right, and you’re not wanting to get it wrong, and you’re conscious that everyone seems to be looking at you too. (P35, 18 years).

Many mothers rejected others’ negative comments as an act of refusal. They actively resisted the stereotypes of young mothers to protect their identity as a young breastfeeding mother by intentionally demonstrating agency and gaining back their power in decision-making around feeding.

I just knew that this is the best thing for him. And I think one of the big, big factors is that, because I was a young single mother when I had him, I didn’t want to fall into the stereotype or the stigma behind it. Like, generally, a lot of young mums do not breastfeed, and I was just adamant to breastfeed for that reason. I’m still really proud of myself for breastfeeding. (P12, 21 years).

There was a whole lot of really unhelpful older mums… who sort of said, ‘You’ll never be able to do this.’ I was 24 when we had [second baby] and… initially there just wasn’t, wasn’t much [support and encouragement], so I sort of thought, ‘It will be too hard.’ But I don’t like being told that I can’t do something, so I’d sort of gone in with the mentality of, ‘Well, I’ll give it a good British try. (P32, 24 years).

Participants managed stigma through avoidance of spaces of stigmatisation. They worked to block threats to their continued breastfeeding actively. They withdrew from people who judged their breastfeeding practice and undermined their goals.

I had a Plunket nurse with our eldest. She was awful. She was the one who basically said I must have been sleeping around, and I deserved every problem that I was getting. I saw her for about six months, and my sister said, ‘Why are you going to see someone who makes you miserable? Why do you have to amp yourself up for a week, knowing that you have to go and see this woman, having a panic attack because you know you’re going to be told you’re doing something wrong? Even if you’re doing nothing wrong, she’ll find something to pick on you about, so why go?’ I didn’t realise you didn’t have to see them. I thought Plunket was a thing that you had to do, and the government was going to come and tell you off if you didn’t do it. I had no idea. (P32, 24 years).

As soon as I walked into the door, I felt judged. And that feeling was strong enough for me to decide that I didn’t value anything she had to say. So I didn’t go there any longer. I stopped going. And that feeling of being judged, that was the reason I didn’t take that advice on. (P3, 24 years).

Oh my god, I can still picture her [Plunket nurse]. Her name was […], and she came in, and she said, ‘Ok, so let’s have a look at the feeding.’ And she kind of looked at my nipples, and I was just saying, ‘You know, it’s still really sore.’ And she just looked at me, and she said, ‘You’re not going to be able to feed. You should put her on the bottle.’ And I just looked at her and thought, ‘Fuck you.’ And basically, she sort of stayed out her visit. She left, and I never let her in the house again. I used to hide behind the couch. She’d come and bang on the door. I mean, I was young, and they obviously thought that I didn’t have a clue. (P27, 23 years).

### Accessing support online

#### Timely and convenient

Participants described the support received online as fast, practical and convenient. Most mothers in our study reported accessing breastfeeding support through the use of private and public Facebook groups. Within these, the timely nature of support requested by the mothers was valued and significant in supporting breastfeeding and reducing a sense of isolation. They could seek and access information quickly and learn tools to help.

When I got mastitis, I had no idea what I was doing. I’d heard of it, but I didn’t really realise that I had mastitis. I just posted on this page, ‘I have these symptoms’, and they were like, ‘Yep, you’ve got mastitis. You need to do this, this, this and this.’ And it was instant information straight away, and I managed to get over the mastitis without taking antibiotics. And the information that came straight away from people who had been through it. (P13, 23 years).

On the Facebook group, within about 30 s, you’ve met 50 women in the local area… it’s support there in the middle of the night, whereas before, it just wasn’t there. You’re sat there, ‘What do I do?’ At least now, you can find someone who may have a suggestion you could try, whereas before, it would be waiting the six, seven hours until something opened at nine and then trying to be brave enough to go and ask for help. (P32, 24 years).

#### Anonymity

Mothers appreciated the anonymous nature of online support as an essential factor for overcoming challenges presented to them. In the context of experiences of stigma and discrimination and erosion of trust in health professionals, the mothers turned to online sources to avoid negative interactions. Accessing breastfeeding information and guidance online was a strategy to minimise judgement and feelings of shame, where they had faced difficulties in open discussion and accessing in-person support previously. In online spaces, their identity was concealed.

It’s good in a respect that you’ve got somewhat anonymous support, especially if it’s things that you’re struggling with and you’re embarrassed about it. (P32, 24 years).

All the questions that people post [on breastfeeding social media page] and all the mums that give answers, that was quite cool ‘cos you don’t actually have to say anything. Like most of the time, if you have a question, it will be answered in there, and you don’t have to feel the embarrassment of telling someone. Whatever problem you’ve got, it’s normally already there. (P15, 18 years).

#### Wealth of experience

Engaging with online Facebook communities enabled the mothers to access a wealth of experience from other mothers. The participants felt they could freely ask a range of questions to access the lived experiences, knowledge and understandings of numerous other mothers. The complexity of breastfeeding challenges was acknowledged, and they valued the range of solutions offered by mothers who had been through similar challenges. The advice provided reassurance that was not accessible to them in other spaces. The online communities were open spaces for learning and sharing.

I think social media works best. A lot of people don’t want to go to health professionals… But if you have the online thing, you’ve got mums from all, you know, they’ve done everything. You can get the advice from everyone. (P8, 23 years).

The Facebook group was a big help in knowing what’s normal for babies, ‘cos with your first, you really don’t know. I researched everything I could, but I still didn’t know that much. And just having the other mums, and because the Facebook groups are not just for first-time mums, there are mums who are on their 2nd, 3rd, 4th, 5th, 10th baby, they’ve got a lot of experience as to what is normal. When you go, ‘My baby won’t sleep more than two or three hours at night, and I’m always having to wake up to feed baby,’ they can go, ‘That is normal for a little baby. No, you don’t have to give them a formula feed before bed, and no, that might actually not help anyway, but if you want to, you can. But if you want to keep breastfeeding, you’re best to pump at the same or a similar time in order to keep your milk supply up. (P33, 23 years).

Stuff about blisters and blocked ducts and supply. At one point, I umm’d and ahh’d, and I was so desperate I was considering medication. But they came up with other ideas, breastfeeding recipes and that old wise women information that would normally be shared over the fence. (P31, 21 years).

#### Social connection and shared values

Online, the young mothers in our study found other mothers who were like-minded and held shared values and experiences around breastfeeding. Beyond gaining information, they made social connections within communities of breastfeeding mothers. In contrast to their previous experiences in their immediate social contexts, they located other mothers with similar mothering and breastfeeding understandings, which provided counter-narratives to the social norms and stigma they had encountered. The concealed nature of social media offered an ideal nexus of exchange.

I feel like I found my kind of group, like a tribe basically, online. There’s lots of stories, positive stories, and I really think it’s the same with birth; I think I read a lot of positive stories, and it really helps me, and it’s the same with breastfeeding. It’s like, even though people had some challenges, it’s like the positive kind of outweighed it? (P6, 23 years).

The positive nature of online communities was linked to feelings of safety and the creation of supportive spaces and social connections during challenging periods. The participants spoke of receiving realistic advice that did not undermine breastfeeding.

It’s about finding a group that has the same sort of values that you do because there’s not one right way to bring up a baby. But often you find people who share a lot of the same sort of things that you want to do, and that way you can share on there without, you know… If you went onto a general forum and talked about something about co-sleeping, there are people who might be very negative towards it, whereas if you’re talking to everyone else who, whether they co-sleep or not themselves, think it’s a viable thing, then you’re more likely to get non-judgemental support. You could go there and go, ‘Ugh, I’m sick of breastfeeding at the moment.’ And people wouldn’t say, ‘Aww, give up and give it a bottle.’ They know that, actually, you do go through those periods. You don’t want to give up breastfeeding; you want people to give you some ideas to make it easier or better. Or you know, ‘They’re cutting a tooth at the moment; it’s awful.’ Someone might tell you a way to get around, so the cutting of the tooth doesn’t involve chewing on the breast or things like that. It’s not, ‘I want to give up breastfeeding,’ it’s actually, ‘I’ve got this issue at the moment. (P38, 24 years).

I think the joys of that connection of being able to find other moms, like if you can’t have them in your physical community, being able to branch out in the wider community and get what should have been passed down. When you think about it, breastfeeding got broken in the 50s, and it became designer posh to give your kids formula, then we lost all that knowledge. The generation before that knew how to clear mastitis, knew how to increase supply, all that stuff. So I had a mother that just didn’t know what to do. (P31, 21 years).

Speaking to other mums, like keeping that support with other mums. It was not that easy. And reading things. And I joined a couple of Facebook breastfeeding pages that were really good. So that you could sit there and read these things and know that you weren’t by yourself. I had problems for that whole first year; new things kept coming up, over and over, like different things each time. So I felt like I kinda went through all the different things that everyone kept bringing up. (P6, 23 years).

## Discussion

### Stigma and the cultural health capital of young breastfeeding mothers

Our research highlights the experiences, causes and consequences of the stigma faced by young breastfeeding mothers. Our findings reveal a significant issue as all participants reported instances of feeling stigmatised for being a young mother, and they suggested an active discouragement of young mothers from breastfeeding. While acknowledging that some positive encounters may exist, our analysis of the data suggests that such instances were not the norm for these participants. The participants did not prominently highlight positive encounters, and the data presented a scarcity of such.

Negative experiences appeared to stem from the deeply ingrained societal attitudes and expectations surrounding young mothers and breastfeeding practices. The young mothers directly connected stigma, breastfeeding practice, and their status as young mothers. They were acutely aware of the judgement and stigma placed on them for being young mothers within their families and communities, as well as the societal expectation that formula feeding is more ‘normal’ for young mothers [37]. Their narratives mirror previous research findings highlighting uneasy relationships between health professionals and young mothers regarding breastfeeding [37]. The participants also highlight the perpetuation of power differences, stigma, bias and discriminatory practices towards young mothers.

Our study found that young breastfeeding mothers felt unequal in their relationships with health professionals, stemming from the stigma of age and denial of knowledge. Their accounts demonstrate how cultural health capital can be eroded when positioned as naïve or lacking understanding. We found that poor advice rooted in age-related assumptions of breastfeeding ability compromised the participants’ capital and status in their encounters with health professionals. Their motivations and decisions were undermined, denying legitimacy and restricting their capacity. Our research shows how stigma can compromise cultural health capital and progress inequitable relationships. These factors can lead to young mothers’ decisions to withdraw from interactions with healthcare services. This finding reflects SmithBattle’s [36] assertion that health professionals have the power to “shape the teen mothers’ experience, and her confidence, trust and willingness to return for care” (p. 239). Our data also align with existing literature, including studies by SmithBattle [36], Ellis-Sloan [34], and Tomori et al. [49], which collectively demonstrate that stigma significantly contributes to feelings of distrust, avoidance, fear, and shame among young mothers. In particular, our participants’ accounts revealed that the mechanisms by which this stigma is internalised are through feeling undermined and belittled in their breastfeeding journeys.

The emerging literature on mental health and stigma resistance parallels our findings [50–51]. Thoits explains similar processes of stigma related to mental illness and its negative consequences [49]. Drawing on Goffman’s [33] work on stigma, Thoits explains that stigmatised people, whilst not identifying with the discrediting identity, can face difficulties in interpersonal interactions, characterised by risk and anxiety and withdrawal as a coping mechanism [50]. Our findings show similar intersecting layers of stigma in the social marginalisation of young breastfeeding mothers.

In light of these findings, it becomes crucial to align our efforts with the World Health Organisation’s (WHO) recommendations on breastfeeding. Addressing the active discouragement of young mothers from breastfeeding by healthcare professionals and society should be a priority. We recommend that healthcare professionals adopt more supportive and sensitive approaches when engaging young mothers to promote breastfeeding.

### The role of online communities in building cultural health capital

Our findings indicate that online spaces play an important role in providing young mothers access to information and advice. Our participants reported that online Facebook groups were a preferred source of support and helped them overcome perceived barriers to breastfeeding. They valued the convenience and timeliness of online support and described their online communities as safe and encouraging spaces where they could freely ask questions and share experiences. This finding aligns with previous research that has found that online spaces provide breastfeeding women with a sense of community, shared goals and experiences, and emotional connections [23, 24, 26, 29].

Our findings contribute to the growing body of evidence that underscores the importance of peer support in maternal health [4, 7]. The young mothers in our study described how online communities played a role in developing social connections, normalising breastfeeding, and improving their breastfeeding experience and continuation. This finding contrasts with their prior interactions with families and health professionals, where breastfeeding was often not culturally accepted.

Additionally, online communities gave the young mothers in our study more autonomy in decision-making and breastfeeding practice and strengthened their identities as breastfeeding mothers. Regan and Brown explain that when breastfeeding is normalised in one’s social context, the mother is more likely to initiate and continue breastfeeding [13]. Online communities also enabled young mothers to circumvent the stigma and discrimination they faced in other spaces by providing managed anonymity and being easily accessible at their convenience.

In line with Jamie et al.’s findings, we argue that being part of online breastfeeding communities enables young breastfeeding mothers to increase their cultural health capital, which is not available in other spaces [37]. The young mothers in our study withdrew from relationships with health professionals and used online communities, particularly Facebook groups, to build their cultural health capital. They contrasted the advice and support received within these online communities as relevant and devoid of stigmatisation, which had a positive impact on breastfeeding continuation [7]. Additionally, our study observed that some of the young mothers transitioned from being recipients of advice and support to becoming providers of knowledge and support themselves, further illustrating the development of their sense of self and the building of cultural health capital, skills, and understanding. Overall, online communities gave young mothers a sense of purpose and contribution, facilitating the development of a meaningful identity and augmenting their cultural health capital, linking to previous research that has found online fora to be particularly supportive for mothers [52]. Given our participants were from a stigmatised group, it would be interesting to explore how much such positionality contrasts with research on middle-class mothers’ experiences, which points out that online fora are often used to reinforce rather than resist normative mothering practices [53]. The particular use of Facebook groups for online support needs to be contextualised within the broader social media space, which has been found to have ambivalent mental health effects for new mothers [53–55]. Our findings underscore the importance of understanding the contextual nature of social media use in new mothers’ experiences to learn about the positive and negative effects of its use, particularly for marginalised groups, such as young mothers.

The importance of peers in the breastfeeding context cannot be understated. While the effectiveness of breastfeeding interventions remains challenging to determine, interventions involving peer support seem to be the most successful in increasing exclusive breastfeeding rates among young mothers [56]. It is essential to recognise that young mothers are understudied in the context of breastfeeding promotion and support interventions. Future intervention studies need to place greater focus on young mothers or ensure sufficient inclusion of young mothers [50].

### Rejecting stereotypes and resisting stigma

Our research aligns with previous studies that indicate that mothers commonly prefer online breastfeeding support groups as a valuable source of information and advice [23, 26, 28]. Our research found that mothers commonly and increasingly turn to online breastfeeding support groups as a preferred source of information and advice [23, 26, 28]. Participants in our study reported that online groups were vital in overcoming perceived barriers to support and valued the convenience and timeliness of the information they received. These online communities also played a role in normalising breastfeeding and providing young mothers with substantial support through positive relationships [13]. In contrast to the lack of cultural acceptance of breastfeeding in other social interactions, online communities provide a safe and encouraging environment for young mothers to continue breastfeeding [15].

The young mothers in our study described how online communities provided access to information and helped them develop social connections, normalise breastfeeding, and improve their overall breastfeeding experience. This finding is consistent with previous research that shows that online spaces provide breastfeeding women with a sense of community, shared goals, and emotional connections [23, 26], access to a collective of like-minded breastfeeding mothers [24, 26], and a sense of social belonging [29]. Moreover, the young mothers in our study used online communities to counter the stigma they faced as young breastfeeding mothers. They actively removed themselves from a social system that stigmatised and excluded them and used online communities to establish their identities as breastfeeding mothers. Being part of these online communities empowered them to build a positive sense of identity, regain control, and persist in their breastfeeding efforts [51]. The participants used virtual communities to construct a positive identity as a “responsible,” “capable,” and “good” breastfeeding mother, distancing themselves from deficit identity assumptions. They exercised agency and autonomy in withdrawing from spaces that discouraged or were not supportive of breastfeeding and successfully normalised breastfeeding in their own lives.

## Study reflections

This research offers valuable insights into the experiences of young mothers who breastfed exclusively for six months or longer in Aotearoa New Zealand. The use of a qualitative research approach allowed for an in-depth exploration of the challenges, support and successes these young mothers encountered throughout their breastfeeding journeys. The study captured rich and detailed narratives by conducting semi-structured interviews, providing a nuanced understanding of their experiences. One of the study’s strengths is its focus on a specific and often understudied population– young mothers who exclusively breastfed for extended periods. As such, the research sheds light on the complexities and nuances of young mothers’ breastfeeding practices.

However, we note several limitations to consider. Firstly, participant recruitment relied on social media posts within specific local Facebook parenting groups. While this approach facilitated the expressions of interest of 44 young mothers who all participated in the telephone interviews, it may have inadvertently narrowed participation to those active on social media and members of these groups. This potentially limits the generalisability of the findings to a broader population of young mothers. While online communities encompass a range of platforms, the scope of this paper does not allow for an in-depth discussion of differences between platforms. The scope of this article focuses largely on internet-based groups and forums accessed by participants (primarily Facebook groups, both private and public). Further research could investigate potential differences in experiences across various social media platforms and apps designed for parenting and breastfeeding support. Secondly, self-selection and the strong level of engagement and cooperation from the target population could have led to an overrepresentation of mothers with particular experiences, affecting the overall balance of perspectives within the study. Thirdly, the retrospective nature of the interviews may have introduced recall bias, as participants might not accurately recall specific details or events related to their breastfeeding journey.

## Conclusion

Our research highlights the importance of online communities as a tool for young mothers to navigate and resist the societal stigmas surrounding breastfeeding. Our study has identified how young breastfeeding mothers use these online communities to assert their autonomy, gain confidence, and succeed in their breastfeeding journeys. These virtual spaces provide a unique structure that can help counteract the adverse effects of social and historical determinants on breastfeeding rates by fostering a sense of inclusion and support.

Our findings have important implications for the development of breastfeeding promotion strategies for young mothers, as the participants demonstrate the effectiveness of peer support in counteracting the negative impacts of stigma. They also highlight the need for an approach that recognises the complex determinants of breastfeeding success, including cultural, historical, and social contexts, particularly for young mothers.

Our research also sheds light on the experiences of young mothers within the health professional relationship and the effects of stigma and cultural health capital on their engagement and withdrawal from services. As Dodgson notes, “Being aware of how power dynamics affect the situations in which we work is essential to understanding social justice issues within the field of lactation” [57]. Therefore, we must continue to consider the sociocultural barriers to breastfeeding that can stigmatise and marginalise young mothers and reflect on their socio-political and economic positioning and how it can exacerbate inequities.

## Data Availability

The datasets used and/or analysed during the current study are available from the corresponding author on reasonable request.

## Footnotes

1 Plunket is New Zealand’s largest provider of the Well Child Tamariki Ora programme, encompassing a series of health visits and support that are free to all families for children from around six weeks up to five years of age.
